# Bidirectional transitions of sarcopenia states in older adults: The longitudinal evidence from CHARLS

**DOI:** 10.1002/jcsm.13541

**Published:** 2024-07-12

**Authors:** Ya‐Xi Luo, Xiao‐Han Zhou, Tian Heng, Ling‐Ling Yang, Ying‐Hai Zhu, Peng Hu, Xiu‐Qing Yao

**Affiliations:** ^1^ Department of Rehabilitation The Second Affiliated Hospital of Chongqing Medical University Chongqing China; ^2^ Chongqing Municipality Clinical Research Center for Geriatric Medicine Chongqing China; ^3^ Department of Rehabilitation Therapy Chongqing Medical University Chongqing China

**Keywords:** Multi‐state Markov model, Older adults, Sarcopenia, States transition

## Abstract

**Background:**

Sarcopenia, the age‐related loss of muscle mass and function, brings multiple adverse outcomes including disability and death. Several sarcopenia consensuses have newly introduced the premorbid concept of possible sarcopenia and recommended early lifestyle interventions. Bidirectional transitions of premorbid states have been revealed in several chronic diseases yet not clarified in sarcopenia. This study aims to investigate the underlying transition patterns of sarcopenia states.

**Methods:**

The study utilized three waves of data from a nationally representative survey, the China Health and Retirement Longitudinal Study (CHARLS), and included community‐dwelling individuals aged 60 years and older with at least two sarcopenia states assessments based on the Asian Working Group for Sarcopenia criteria 2019 (AWGS2019) between 2011 and 2015. The estimated transition intensity and probability between non‐sarcopenia, possible sarcopenia, sarcopenia, and death were investigated using multi‐stage Markov (MSM) models.

**Results:**

The study comprised 4395 individuals (49.2% female, median age 67 years) with a total of 10 778 records of sarcopenia state assessment, and the mean follow‐up period was 3.29 years. A total of 24.5% of individuals with a current state of possible sarcopenia returned to non‐sarcopenia, 60.3% remained possible sarcopenia, 6.7% progressed to sarcopenia, and 8.5% died by the next follow‐up. The transition intensity of recovery to non‐sarcopenia (0.252, 95% CI 0.231–0.275) was 2.8 times greater than the deterioration to sarcopenia (0.090, 95% CI 0.080–0.100) for individuals with possible sarcopenia. For individuals with possible sarcopenia, the estimated probabilities of recovering to non‐sarcopenia, progressing to sarcopenia, and transitioning to death within a 1‐year observation were 0.181, 0.066, and 0.035, respectively. For individuals with sarcopenia, the estimated probabilities of recovering to non‐sarcopenia, recovering to possible sarcopenia, and transitioning to death within 1‐year observation were 0.016, 0.125, and 0.075, respectively. In covariables analysis, age, sex, body mass index, physical function impairment, smoking, hypertension, and diabetes are important factors influencing bidirectional transitions.

**Conclusions:**

The findings highlight the bidirectional transitions of sarcopenia states among older adults and reveal a notable proportion of possible sarcopenia show potential for recovery in the natural course. Screening and intensifying interventions based on risk factors may facilitate a recovery transition.

## Introduction

Sarcopenia, an age‐related disease characterized by progressive loss of skeletal muscle mass (SMM) and functions,^1^ has been proven to increase the risk of falls and fractures, lead to loss of independence and the need for long‐term care or even death, resulting in a high social and economic burden.^2^, ^3^ As the world ages, the prevalence of sarcopenia is expected to rise in parallel [S1]. The most commonly used diagnostic criteria for sarcopenia is consensus developed by the European Working Group on Sarcopenia in Older People (EWGSOP) and updated in 2019.^2^ The diagnosis of sarcopenia in Asian populations is usually based on an adapted diagnostic consensus for Asians by the Asian Working Group on Sarcopenia (AWGS).^4^ The concept of possible sarcopenia, a premorbid state between non‐sarcopenia and sarcopenia, was introduced in the latest updates of both consensuses.

This concept of premorbid state also exists in diseases such as Alzheimer's disease^5^ and diabetes mellitus [S2], where the premorbid state has a bidirectional transition to recovery or progression. Several studies have shown that lifestyle interventions including resistance exercise and optimizing nutritional intake may help improve sarcopenia conditions^6^ [S3,S4]. Sarcopenia is a chronic disease that evolves dynamically, giving rise to varied developmental trajectories that individuals may encounter. However, the mutual transitions between the normal, premorbid, and disease states of sarcopenia remain unclear.

In recent years, multi‐state Markov (MSM) models have been applied in the health field to analyse the mutual state transitions of chronic diseases including Alzheimer's disease^5^ and hypertension.^7^ MSM model can demonstrate the multi‐state process by transition intensity between different states, dynamically predict the probability of transitions between different disease states, and estimate the effects of covariates on transitions and the sojourn time of individuals in specific disease states. There is a lack of clarity regarding transitions between sarcopenia states. This study analyzes data from a nationally representative longitudinal survey of China's population of older adults using the MSM model, with the aim of revealing the progression and fluctuating course of sarcopenia and providing information on the transition probability between sarcopenia states, effects of covariates on transitions, estimated mean sojourn times and total stay in sarcopenia states.

## Methods

### Study population

The population for this study was derived from the China Health and Retirement Longitudinal Study (CHARLS), a nationally representative longitudinal survey initiated in 2011. CHARLS aims to investigate the social, economic, and health status of community residents aged 45 years and older in China.^8^ The survey covered 450 villages, 150 counties, and 28 provinces, with four survey waves released to date (Wave 1, 2011; Wave 2, 2013; Wave 3, 2015; and Wave 4, 2018). The CHARLS study randomly selected 150 counties from the 28 provincial administrative units in China using probability proportional to size sampling, resulting in a final sample size of over 17 000 individuals at baseline. All interviewers of the survey were trained at Peking University by CHARLS staff members and conducted surveys strictly according to the survey manual using a Computer‐Assisted Personal Interview (CAPI) system. Detailed information about the CHARLS has been previously published,^8^ and data are available for download at http://CHARLS.pku.edu.cn/en. The CHARLS was approved by the Biomedical Ethics Review Committee of Peking University (IRB00001052‐11015). All participants signed informed consent. The present study was conducted following the Strengthening the Reporting of Observational Studies in Epidemiology (STROBE) reporting guideline [S5].

**Figure 1:**
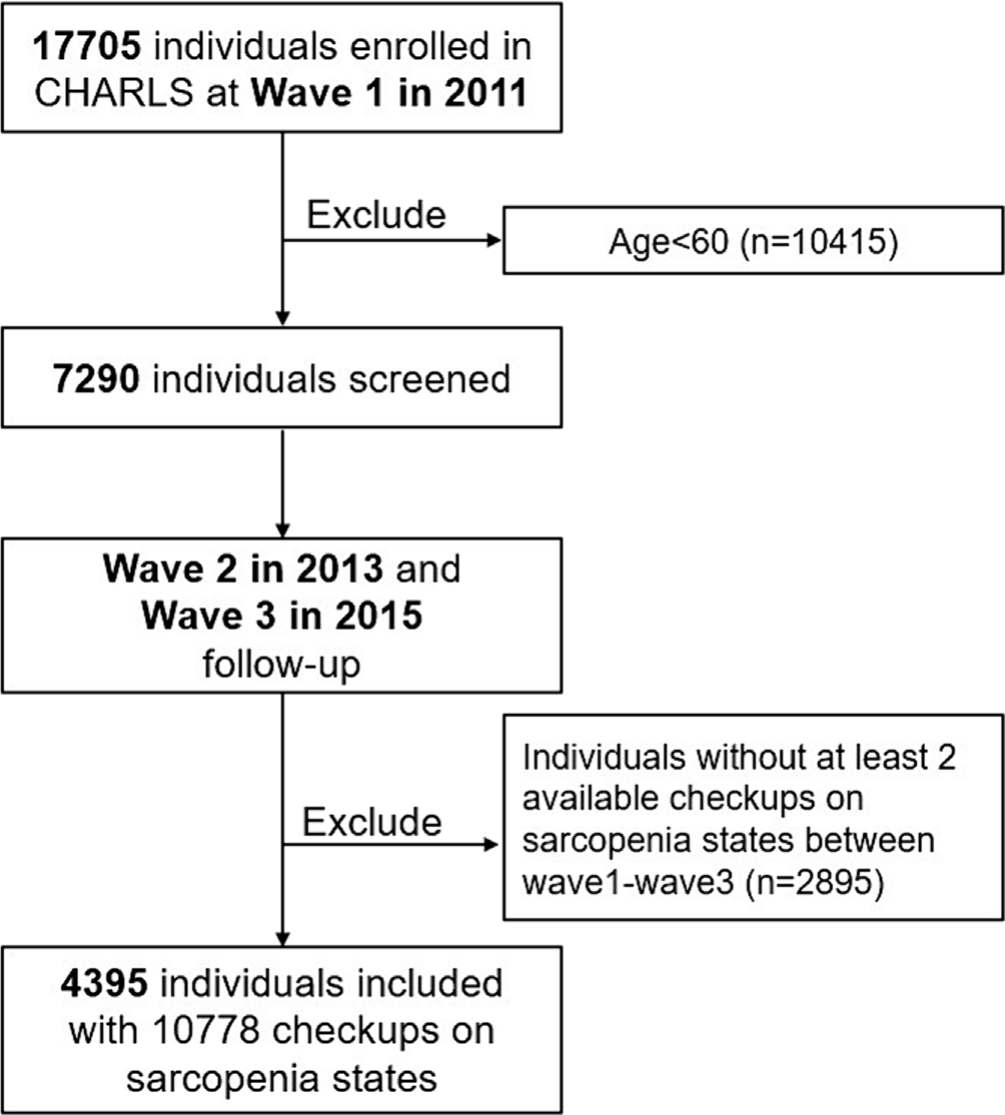
The flow chart of screening for enrolled individuals.

Of data in four waves of CHARLS, Wave 1–Wave 3 contained anthropometric and functional performance assessments necessary to determine sarcopenia states, so data from Wave 1–Wave 3 were included. The exclusion criteria are as follows: (1) age less than 60 years and (2) the unavailability of data for at least two sarcopenia state assessments required for state transitions evaluation. The detailed process of screening was shown in Figure 1. The comparison of characteristics between enrolled and excluded individuals lacking follow‐up was described in Table S1.

### Diagnosis of sarcopenia states

According to the AWGS2019,^4^ normal muscle strength and a normal chair test were determined as non‐sarcopenia. Possible sarcopenia was defined as the presence of low muscle strength or physical performance evaluated through the chair stand test but did not yet meet the sarcopenia diagnostic criteria. Sarcopenia is defined as low muscle mass combined with low muscle strength or low physical performance.

Grip strength was used to evaluate skeletal muscle strength. Trained examiners conducted the measurement with the use of a Yuejian TM WL‐1000 dynamometer in kilograms (Nantong Yuejian Physical Measuring Instruments Co., Ltd., Nantong), and the average of the maximum available values for two repeated measurements were adopted [S6]. A cutoff point of <18 kg for women and <28 kg for men as the threshold for low muscle strength was implemented.^4^

Measurement of physical performance includes gait speed, chair stand test, and the short physical performance battery (SPPB) test [S7]. The criteria for low physical performance are gait speed < 1.0 m/s, 5 chair stand tests ≥ 12 s, or SPPB ≤ 9.^4^

Muscle mass was estimated by appendicular skeletal muscle mass (ASM) using a previously validated and applied anthropometric equation^9^, ^10^ and the strong agreement between the ASM obtained through this equation and the one derived from dual‐energy X‐ray absorptiometry (DXA) in the Chinese population has been proven.^11^

$$\mathrm{ASM}=0.193*\text{weight}\left(\mathrm{kg}\right)+0.107*\text{height}\left(\mathrm{cm}\right)-4.157*\mathrm{sex}\left(1\;\text{for male},2\;\text{for female}\right)-0.037*\mathrm{age}\left(\mathrm{yr}\right)-2.631$$

Height was measured using a SecaTM213 stadiometer (Seca Trading, Hangzhou, China). Weight was measured using an Omron™ HN‐286 scale [Krell Precision (Yangzhou) Co. LTD., Yangzhou, China]. The height‐adjusted muscle mass (ASM/Ht^2^) was calculated by dividing the ASM value by the square meters of height. The cutoff value for defining low muscle mass was based on the sex‐specific lower 20th percentile of ASM/Ht^2^ in the study population,^9^, ^10^ which is 4.90 kg/m^2^ for females and 6.79 kg/m^2^ for males in the present study.

### Covariates

Covariates included age, sex, marital status, education, physical function, depression symptoms, smoking, alcohol consumption, body mass index (BMI), and chronic disease conditions. Physical function was evaluated by a 6‐item activities of daily living (ADL) scale, and individuals reported some difficulty in performing tasks including dressing, bathing, eating, getting in and out of bed, using the toilet, and controlling urination and defecation. The total score ranged from 0 to 6, with a score of 0 indicating no impairment, 1–2 representing mild impairment, and 3 or more indicating severe impairment [S8]. Depressive symptoms were evaluated through the Center for epidemiologic studies depression scale (CESD‐10), where a score of <10 indicates the absence of depressive symptoms while a score of ≥10 suggests the presence of depressive symptoms.^12^ BMI is classified into three categories: underweight (BMI < 18.5 kg/m^2^), normal weight (18.5 kg/m^2^ ≤ BMI < 24 kg/m^2^), and overweight/obese (BMI ≥ 24 kg/m^2^) according to Chinese classification.^13^ Chronic disease conditions include hypertension, diabetes mellitus, cardiac disease, pulmonary disease, history of stroke, and cancer diagnosed by doctors. We quantified the number of chronic co‐morbidities and categorized them into three groups: ‘no condition’, ‘1 condition’, and ‘2 or more conditions’. Missing data are described in Table S2. Multiple imputation was used to fill in the missing values for subsequent analysis.

### MSM models and statistical analysis

**Figure 2:**
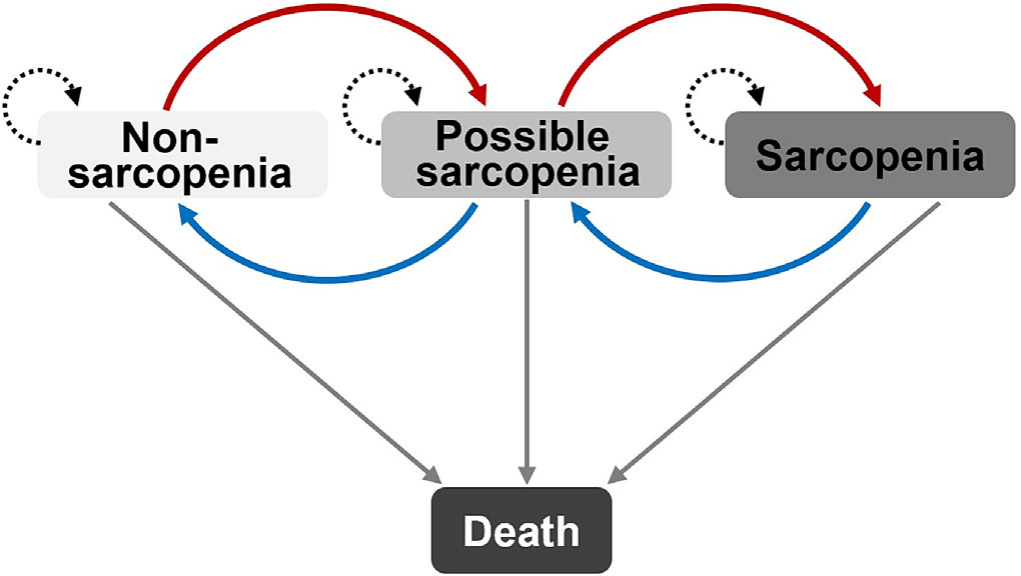
The hypothesis of transitions between sarcopenia states. Non‐sarcopenia, possible sarcopenia, and sarcopenia are designated as transient states while death is the absorbing state. Transient states can move between adjacent states, but once an absorbing state is reached, no further transitions can occur.

MSM package within R 4.0.0 was used to construct MSM models in continuous time.^14^ MSM is a package of functions for multi‐state modelling that performs maximum likelihood estimation in continuous time for general MSM models. We defined four possible states (non‐sarcopenia marked as state 1, possible sarcopenia marked as 2, sarcopenia marked as 3, and death marked as 4), where non‐sarcopenia, possible sarcopenia, and sarcopenia are designated as transient states while death as the absorbing state (Figure 2). Transient states can move between adjacent states, but once an absorbing state is reached, no further transitions can occur. Individuals can advance or recover between adjacent disease states, or die from any state. The Markov assumption claims that the rate of transition from one state to another depends only on the current state. The transition intensity matrix, which determines these transitions, is denoted as Q and is given below:

$$Q=\left(\begin{array}{cccc} -\left(q_{12}+q_{14}\right) & q_{12} & 0 & q_{14}\\ q_{21} & -\left(q_{21}+q_{23}+q_{24}\right) & q_{23} & q_{24}\\ 0 & q_{32} & -\left(q_{32}+q_{34}\right) & q_{34}\\ 0 & 0 & 0 & 0 \end{array}\right)$$

The meaning of qrs is the instantaneous rate from state r to s. Let x(t) = r denote the state time t of r. The transition intensity of the individual is calculated for the time interval (t, t + t) in which the move to state s (s = 1, 2, 3, 4).

$$q_{\mathrm{rs}}\left(t\right)=\lim_{\mathrm{\delta t}\to 0}P\left(x\left(t+\mathrm{\delta t}\right)=s|x\left(t\right)=r\right)/\mathrm{\delta t}$$

The qrs form a 4 × 4 matrix Q whose rows sum to zero, and the diagonal entries are defined by $q_{\mathrm{rr}}=-\sum_{s\neq r}q_{\mathrm{rs}}$. Transitioning instantaneously from non‐sarcopenia to sarcopenia is unrealistic due to the prolonged and gradual nature of sarcopenia, so q13 and q31 are assigned a value of 0. As death is an absorbing state q41, q42, q43, and q44 are also set to 0. The process of fitting the model involves determining the seven unknown transition intensities (q12, q14, q21, q23, q24, q32, and q34) based on Markov assumptions with the quasi‐Newton method and maximizes the likelihood.

The probability that an individual in state r moves next to state *s* is $-q_{\mathrm{rs}}/q_{\mathrm{rr}}$. Mean sojourn times describe the average period in a single stay in a state before the next state transition occurs. Based on Markov assumption, in a time‐homogeneous Markov model, in which qrs are independent of *t*, the sojourn time in each state *r* is exponentially distributed with mean $-1/{\hat{q}}_{\mathrm{rr}}$, where ${\hat{q}}_{\mathrm{rr}}$ is the r th diagonal entry of the estimated transition intensity matrix. The predicted total length of stay in each transient state s between two future time points t1 (time 0, the present time) and t2 (death) and was obtained as $L_{S}=\int_{t1}^{t2}P{\left(t\right)}_{r,s}\mathrm{dt}$. A univariate MSM model was conducted to examine the effects of covariates on state transitions. We further established a multivariate MSM model that included covariates that significantly affected bidirectional transitions of sarcopenia states and estimated the transition probability and sojourn time and total stay in subgroups. Statistical analyses were performed using R 4.0.0 (statistical and computing software; http://www.r‐project.org/). A two‐tailed *P* < 0.05 was considered statistically significant.

## Results

### Baseline characteristics of enrolled individuals

A total of 4395 individuals were included, 49.2% were female, and the median age was 67 years (IQR 63–73 years). The baseline sarcopenia states of the participants were as follows: 1628 (37.0%) individuals were non‐sarcopenia, 2014 (45.8%) individuals were possible sarcopenia, and 753 (17.1%) individuals were sarcopenia. The distribution of sarcopenia states in each wave was summarized (Table S3). The baseline demographic and clinical characteristics of these individuals are summarized (Table 1).

**Table 1:** Baseline demographic and clinical characteristics of enrolled individuals

Demographic and clinical characteristics (*n*, %)		Enrolled individuals (*N* = 4395)
Age	60–70	2935 (66.8)
	71–80	1212 (27.6)
	>80	248 (5.6)
Sex	Female	2161 (49.2)
	Male	2234 (50.8)
Marital status	Married/partnered	3430 (78.0)
	Divorced/separated/widowed/never married	965 (22.0)
Education	Below high school level	4203 (95.6)
	At or above high school level	192 (4.4)
Body mass index	Normal	2401 (54.6)
	Underweight	497 (11.3)
	Overweight and obese	1497 (34.1)
Physical function	No impairment	3262 (74.2)
	Mild impairment	787 (17.9)
	Severe impairment	346 (7.9)
Depressive symptoms	No	2549 (58.0)
	Yes	1846 (42.0)
Smoking	No	3114 (70.9)
	Yes	1281 (29.1)
Alcohol consumption	No	3054 (69.5)
	Yes	1341 (30.5)
Hypertension	No	2921 (66.5)
	Yes	1474 (33.5)
Diabetes mellitus	No	4072 (92.7)
	Yes	323 (7.3)
Pulmonary diseases	No	3761 (85.6)
	Yes	634 (14.4)
Cardiac diseases	No	3726 (84.8)
	Yes	669 (15.2)
Cancer history	No	4355 (99.1)
	Yes	40 (0.9)
Stroke history	No	4208 (95.7)
	Yes	187 (4.2)
Number of co‐morbidities	No condition	2098 (47.7)
	1 condition	1497 (34.1)
	2 or more conditions	800 (18.2)

### Transitions between sarcopenia states

The mean follow‐up time was 3.29 years (SD 1.01 years), with 2407 individuals having 1 sarcopenia state follow‐up and 1988 individuals having 2 state follow‐ups. This resulted in a total of 10 778 sarcopenia state assessment records. By the last follow‐up, 1526 (34.7%) individuals were non‐sarcopenia, 1685 were possible sarcopenia (38.3%), 683 (15.5%) were sarcopenia, and 501 (11.4%) died.

The observed transitions from the current state to the next state at follow‐up were summarized (Table S4). For those with a current state of possible sarcopenia, 24.5% returned to non‐sarcopenia, 6.7% progressed to sarcopenia, and 8.5% progressed to death at the next follow‐up. For those with a current state of sarcopenia, 11.1% recovered to possible sarcopenia, 14.3% recovered to non‐sarcopenia, and 14.6% progressed to death at the next follow‐up. For those with a current state of non‐sarcopenia, 27.2% progressed to possible sarcopenia, 7.2% progressed to sarcopenia, and 4.2% transited to death at the next follow‐up.

The transition intensity between sarcopenia states was shown in Table 2. For individuals with possible sarcopenia, the transition intensity of recovery to non‐sarcopenia (0.252, 95% CI 0.231–0.275) was 2.8 times greater than the transition intensity of deteriorating to sarcopenia (0.090, 95% CI 0.080–0.100). The transition intensity of deteriorating to possible sarcopenia from non‐sarcopenia (0.303, 95% CI 0.279–0.330) was 1.8 times greater than the transition intensity of deteriorating to sarcopenia from possible sarcopenia (0.168, 95% CI 0.148–0.191).

**Table 2:** Transition intensity between different sarcopenia states

Transition categories	Transition intensity (95% CI)
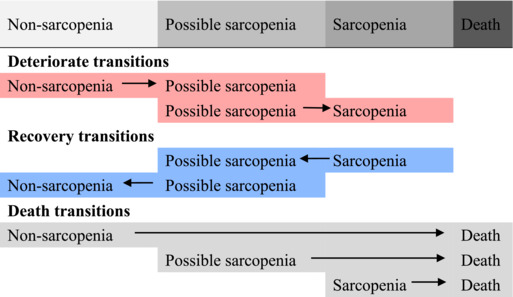	
	0.303 (0.279, 0.330)
	0.090 (0.080, 0.100)
	0.168 (0.148, 0.191)
	0.252 (0.231, 0.275)
	0.010 (0.005, 0.019)
	0.037 (0.029, 0.046)
	0.082 (0.068, 0.098)

### Probability of sarcopenia state transitions

**Figure 3:**
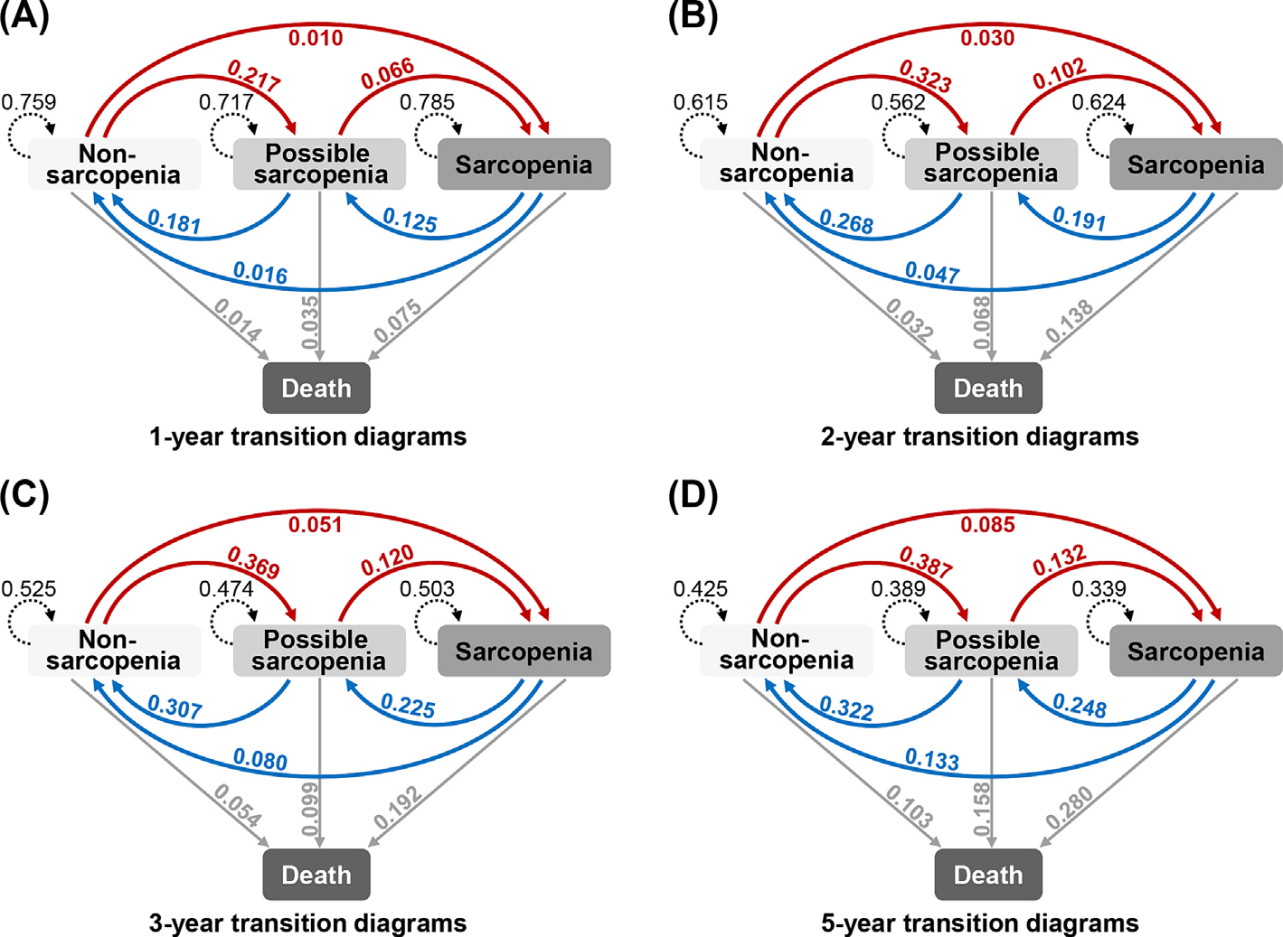
Transition diagrams of sarcopenia states within 1, 2, 3, and 5‐year observation intervals. The state transition diagrams for 1‐year (A), 2‐year (B), 3‐year (C), and 5‐year (D) observation intervals based on the MSM model, respectively. To be specific, in (A), for individuals in possible sarcopenia states, the estimated probability of observing maintaining the current state (dashed line from the box of possible sarcopenia), recovery to non‐sarcopenia (blue line from the box of possible sarcopenia) and progression to sarcopenia (red line from the box of possible sarcopenia), or death (grey line from the box of possible sarcopenia) within 1 year was 0.717, 0.181, 0.066, and 0.035, respectively.

The probability of state transitions during observation intervals of 1, 2, 3, and 5 years for individuals with different sarcopenia states was estimated (Figure 3). For individuals with possible sarcopenia, the estimated probabilities of observing a recovery to non‐sarcopenia, remaining as possible sarcopenia, deteriorating to sarcopenia, and transitioning to death were 0.181, 0.717, 0.066, and 0.035 within a 1‐year observation and evolved into 0.322, 0.389, 0.132, and 0.158 within a 5‐year observation, respectively. For individuals with sarcopenia, the estimated probabilities of observing a recovery to non‐sarcopenia, recovery to possible sarcopenia, remaining as sarcopenia, and transitioning to death was 0.016, 0.125, 0.785, and 0.075 within a 1‐year observation and evolved into 0.133, 0.248, 0.339, and 0.280 within a 5‐year observation, respectively. For individuals with non‐sarcopenia, the estimated probabilities of observing remaining as non‐sarcopenia, deteriorating to possible sarcopenia, deteriorating to sarcopenia, and transitioning to death were 0.759, 0.217, 0.010, and 0.014 within a 1‐year observation and evolved into 0.425, 0.387, 0.085, and 0.103 within a 5‐year observation, respectively.

### Covariate effects on sarcopenia transitions

The effects of covariates on the hazard ratios (HRs) of transitions between different sarcopenia states were estimated in Table 3. Age was associated with worsening, recovery, and death transitions in sarcopenia states. HRs for worsening transitions were significantly higher in both the 71–80 years group (0.169, 95% CI 1.330–2.151) as well as the >80 years group (1.600, 95% CI 1.014–2.524) compared with the 60–70 years group. The likelihood of recovery transition of sarcopenia states decreased with age, with HRs of 0.448 (95% CI 0.343–0.585) and 0.513 (95% CI 0.410–0.642) for transitions from sarcopenia to possible sarcopenia and from possible sarcopenia to no sarcopenia in the group of 71–80 years, respectively, while only HRs of 0.279 (95% CI 0.165–0.471) and 0.106 (0.046–0.246) for the group above 80 years, respectively.

**Table 3:** Effects of the covariate on transitions among sarcopenia states transitions

Hazard Ratio (95% CI)							
**Covariates**	**Deteriorate transition**		**Recovery transition**		**Death transition**		
	**Non‐sarcopenia→ Possible sarcopenia**	**Possible sarcopenia→ Sarcopenia**	**Sarcopenia→ Possible sarcopenia**	**Possible sarcopenia→ Non‐sarcopenia**	**Non‐sarcopenia→ Death**	**Possible sarcopenia →Death**	**Sarcopenia→ Death**
**Age**							
60‐70	Reference		Reference		Reference		
71‐80	**1.473 (1.206‐1.798)**	**1.691 (1.330‐2.151)**	**0.448 (0.343‐0.585)**	**0.513 (0.410‐0.642)**	1.914 (0.507‐7.223)	1.227 (0.687‐2.192)	**4.344 (1.778‐10.614)**
>80	1.487 (0.754‐2.929)	**1.600 (1.014‐2.524)**	**0.279 (0.165‐0.471)**	**0.106 (0.046‐0.246)**	1.812 (0.002‐1333.306)	**4.768 (2.933‐7.751)**	**6.332 (2.528‐15.859)**
**Sex**							
Male	Reference		Reference		Reference		
Female	1.116 (0.940‐1.323)	**0.715 (0.570‐0.898)**	**0.772 (0.600‐0.994)**	**0.795 (0.667‐0.949)**	0.142 (0.001‐18.363)	0.639 (0.405‐1.007)	0.722 (0.497‐1.047)
**Marital status**							
Married/partnered	Reference		Reference		Reference		
Divorced/separated/widowed/never married	1.029 (0.836‐1.267)	0.974 (0.742‐1.279)	0.916 (0.681‐1.233)	0.904 (0.728‐1.124)	0.947 (0.163‐5.515)	0.792 (0.422‐1.487)	1.214 (0.815‐1.808)
**Education**							
Below high school level	Reference		Reference		Reference		
At or above high school level	0.815 (0.526‐1.263)	1.744 (0.950‐3.200)	1.872 (0.968‐ 3.624)	1.620 (0.979‐2.680)	0.911 (0.099‐ 8.372)	1.465 (0.603‐3.564)	0.087 (0.001‐15.591)
**Body mass index**							
Normal	Reference		Reference		Reference		
Underweight	**1.976 (1.298‐3.009)**	**36.356 (13.600‐97.191)**	**3.174 (1.442‐6.985)**	**6.742 (4.028‐11.286)**	0.386 (0.001‐603.185)	**20.407 (12.090‐34.442)**	0.032 (0.001‐58.082)
Overweight/obese	**0.831 (0.701‐0.986)**	**0.320 (0.221‐0.464)**	0.026 (0.001‐65.163)	**0.825 (0.691‐0.985)**	1.432 (0.330‐6.217)	**0.312 (0.100‐0.976)**	**12.734 (7.860‐20.630)**
**Physical function**							
No impairment	Reference		Reference		Reference		
Mild impairment	**1.415 (1.120‐1.788)**	0.598 (0.438‐20.816)	**0.624 (0.444‐0.876)**	**0.720 (0.563‐0.920)**	1.952 (0.496‐7.687)	1.460 (0.807‐2.642)	1.110 (0.722‐1.707)
Severe impairment	**1.795 (1.204‐2.677)**	1.208 (0.832‐1.752)	0.693 (0.409‐1.173)	**0.579 (0.374‐0.894)**	2.386 (0.262‐21.749)	**2.867 (1.611‐5.104)**	**1.940 (1.162‐3.241)**
**Depressive symptoms**							
No	Reference		Reference		Reference		
Yes	**1.397 (1.160‐1.683)**	0.881 (0.701‐1.106)	0.950 (0.738‐1.224)	0.842 (0.698‐1.015)	0.910 (0.136‐6.102)	1.395 (0.860‐2.264)	0.710 (0.485‐1.039)
**Smoking**							
No	Reference		Reference		Reference		
Yes	0.885 (0.729‐1.076)	**1.985 (1.552‐2.538)**	**1.618 (1.234‐2.122)**	1.189 (0.973‐1.451)	**5.245 (1.232‐22.330)**	0.728 (0.366‐1.448)	1.185 (0.795‐1.766)
**Alcohol consumption**							
No	Reference		Reference		Reference		
Yes	0.918 (0.763‐1.106)	1.072 (0.831‐1.381)	1.139 (0.864‐1.501)	**1.410 (1.160‐1.712)**	1.845 (0.511‐6.656)	0.910 (0.511‐1.619)	0.683 (0.424‐1.102)
**Hypertension**							
No	Reference		Reference		Reference		
Yes	0.883 (0.739‐1.056)	**0.469 (0.362‐0.609)**	0.981 (0.719‐1.337)	**0.559 (0.465‐0.672)**	2.930 (0.756‐11.350)	1.571 (0.975‐2.534)	1.346 (0.885‐2.046)
**Diabetes Mellitus**							
No	Reference		Reference		Reference		
Yes	0.814 (0.607‐1.091)	**0.470 (0.270‐0.819)**	0.802 (0.349‐1.842)	**0.572 (0.416‐0.786)**	1.106 (0.179‐6.835)	1.482 (0.865‐2.537)	1.371 (0.458‐4.106)
**Pulmonary disease**							
No	Reference		Reference		Reference		
Yes	1.131 (0.878‐1.455)	**1.393 (1.038‐1.870)**	0.816 (0.580‐1.146)	0.844 (0.645‐1.103)	1.408 (0.227‐8.724)	1.602 (0.929‐2.761)	1.375 (0.908‐2.081)
**Cardiac disease**							
No	Reference		Reference		Reference		
Yes	1.228 (0.977‐1.543)	0.736 (0.538‐1.007)	0.835 (0.554‐1.257)	0.871 (0.679‐1.117)	0.930 (0.150‐5.776)	**1.727 (1.091‐2.736)**	0.739 (0.383‐1.427)
**Stroke history**							
No	Reference		Reference		Reference		
Yes	1.068 (0.685‐1.666)	0.702 (0.382‐1.289)	0.934 (0.457‐1.911)	0.771 (0.493‐1.206)	**5.602 (1.282‐24.489)**	1.812 (0.831‐3.951)	1.340 (0.559‐3.219)
**Number of comorbidities**							
No condition	Reference		Reference		Reference		
1 condition	0.901 (0.740‐1.096)	0.890 (0.694‐1.143)	0.888 (0.672‐1.175)	**0.730 (0.598‐0.892)**	2.652 (0.636‐11.064)	1.129 (0.523‐2.438)	1.451 (0.987‐2.133)
2 or more conditions	0.994 (0.791‐1.249)	0.480 (0.343‐0.672)	0.783 (0.514‐1.192)	**0.495 (0.387‐0.633)**	2.702 (0.551‐13.260)	**2.734 (1.522‐4.910)**	1.424 (0.838‐2.418)

Boldfaced data indicate statistical significance (*P* < 0.05).

Underweight individuals were more likely to manifest a higher likelihood of both recovery and worsening compared to those of normal weight, with HRs of 1.976 (95% CI 1.298–3.009) and 36.356 (95% CI 13.600–97.191) for transitions from non‐sarcopenia to possible sarcopenia and from possible sarcopenia to sarcopenia, respectively, and were also more likely to manifest recovery transitions compared with the normal group, with HRs of 3.174 (95% CI 1.442–6.985) and 6.742 (95% CI 4.028–11.286) for transitions from sarcopenia to possible sarcopenia and from possible sarcopenia to non‐sarcopenia, respectively.

Mild and severe physical impairments increased the risk of transitions from non‐sarcopenia to possible sarcopenia, with HRs of 1.415 (95% CI 1.120–1.788) and 1.795 (95% CI 1.204–2.677), respectively. Mild physical impairments were detrimental to the recovery transition from sarcopenia to possible sarcopenia (HR 0.624, 95% CI 0.444–0.876) and from possible sarcopenia to non‐sarcopenia (HR 0.720, 95% CI 0.563–0.920).

Other factors including sex, smoking, hypertension, and diabetes also show impacts on the bidirectional transitions of sarcopenia states. The risk of deteriorating transitions from possible sarcopenia to sarcopenia was significantly lower in women compared to men (HR 0.715, 95% CI 0.570–0.898). However, women were also significantly less likely to recover from sarcopenia to possible sarcopenia and from possible sarcopenia to non‐sarcopenia than men, with HR of 0.772 (95% CI 0.600–0.994) and 0.795 (95% CI 0.667–0.949), respectively.

### The transition possibility of possible sarcopenia in subgroups

**Figure 4:**
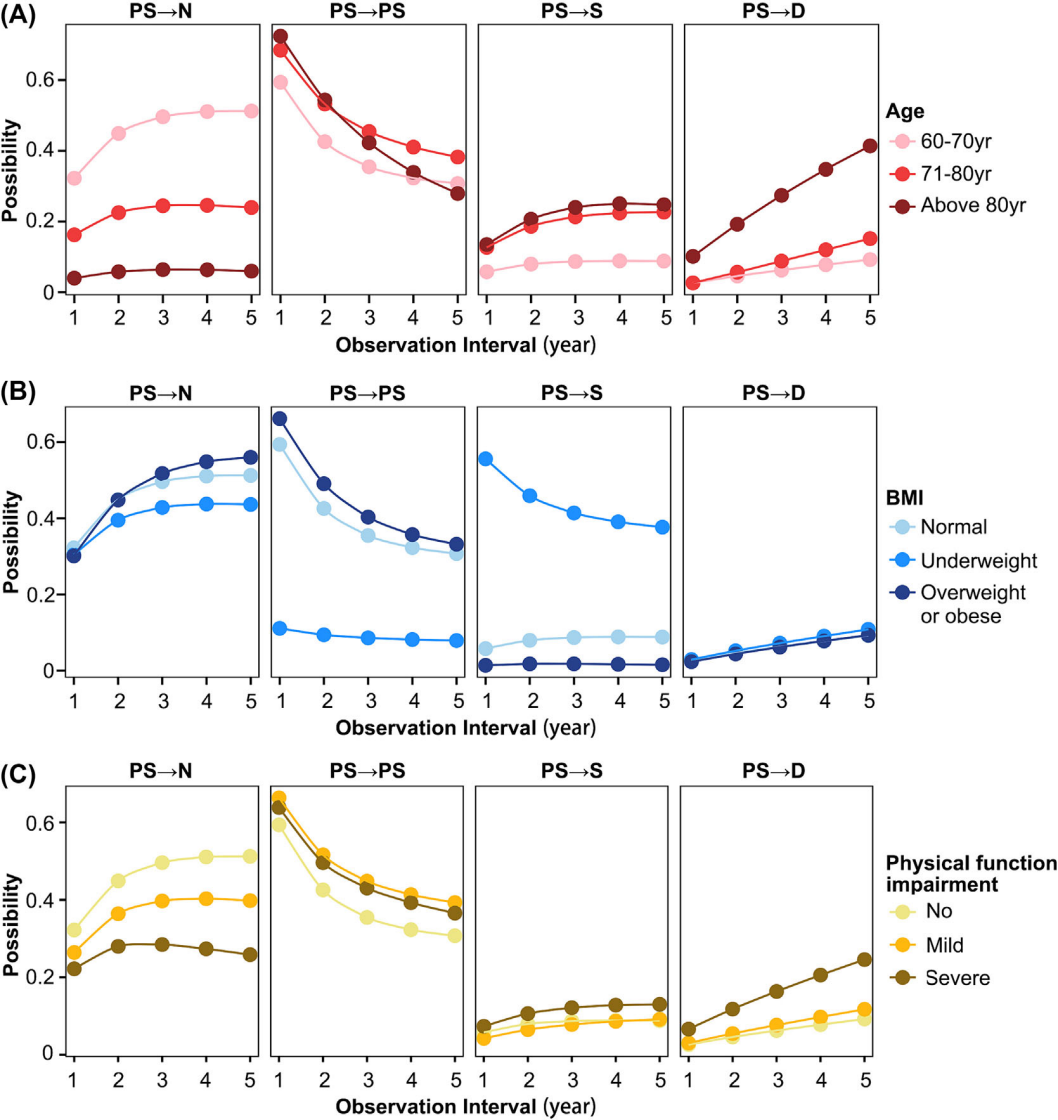
The estimated possibility of possible sarcopenia state transitions within different subgroups. The estimated possibility of possible sarcopenia recovering to non‐sarcopenia, remaining as possible sarcopenia, worsening to sarcopenia, or transitioning to death among age subgroups (A), BMI subgroups (B), and physical function impairment subgroups (C) within 5‐year observation interval, respectively. BMI, body mass index; D, death; N, non‐sarcopenia; PS, possible sarcopenia; S, sarcopenia.

We included covariates that simultaneously affected the deteriorate and recovery transitions of sarcopenia states in a multifactorial MSM model (Table S5) and further estimated the possibility of different transitions from possible sarcopenia among subgroups of age, BMI, and physical function (Figure 4). The possibility of recovery transition from possible sarcopenia to non‐sarcopenia decreases with age. The probability of deterioration of possible sarcopenia was close in the 71–80 years group and the >80 years group, both of which were higher than the 60–70 years group, while the possibility of death transition of possible sarcopenia was highest in the 80 years and older group. The underweight group was less likely to remain unchanged in possible sarcopenia than the non‐sarcopenia and overweight or obese groups, with a higher possibility of worsening transition from possible sarcopenia to sarcopenia at the same time. As the degree of physical function impairment increases, the possibility of transition from possible sarcopenia to non‐sarcopenia decreases.

### Sojourn time and total stay of each transient state

**Figure 5:**
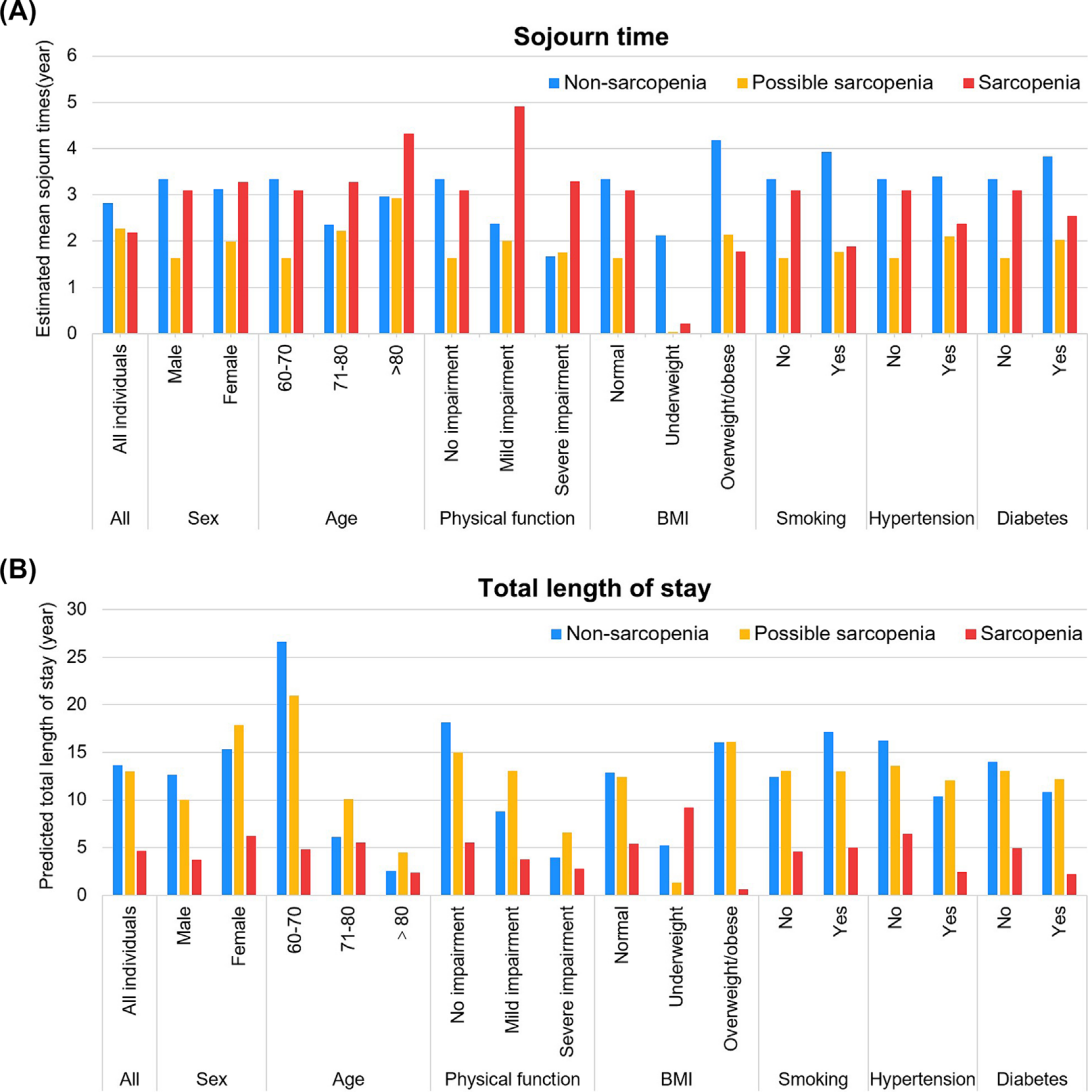
The estimated sojourn time and predicted total stay of sarcopenia transient states. (A) the estimated mean sojourn time of each sarcopenia transient state before the next transition for the overall population and subgroups; (B) the predicted total length of stay in each sarcopenia transient state before death for the overall population and subgroups.

We estimated the mean sojourn time in each transient state and predicted the total stay of each transient state before death in the overall population and subgroups (Figure 5 and Table S6). In the overall population, the mean sojourn time in the non‐sarcopenia, possible sarcopenia, and sarcopenia states were 2.83, 2.27, and 2.19 years, respectively. The estimated total stay of each transient state before death in the non‐sarcopenia, possible sarcopenia, and sarcopenia states were 13.67, 13.02, and 4.67 years, respectively.

## Discussion

The present study clarified the bidirectional transitions in sarcopenia states among a nationally sampled Chinese geriatric population and derived the transition intensity and probabilities. Our results emphasize that the sarcopenia states can transition in both directions among older adults, and a notable proportion of possible sarcopenia show potential for recovery. Important factors including age, BMI, and physical function impairment show an influence on bidirectional transitions.

Sarcopenia was recognized as a disease by the International Classification of Diseases, 10th Revision, Clinical Modification (ICD‐10‐CM) in 2016 [S9], and the premorbid concept of possible sarcopenia was introduced in the recent updates in AWGS 2019^4^ and EWGSOP 2019.^2^ Our result revealed 39.4–46.0% of individuals were diagnosed with possible sarcopenia in Wave 1–Wave 3, while 15.2–17.6% were diagnosed with sarcopenia. The prevalence of possible sarcopenia, as determined by the AWGS 2019 criteria was reported in two previous studies.^15^, ^16^ One study of Asians over 60 years old found 38.9% had possible sarcopenia^15^ and another study of 275 Asian rural older adults, revealed possible sarcopenia in 68.7% of participants.^16^ The former study used grip strength abnormalities solely as the criterion, while the other study had 75% female participants, which may explain the disparity between the results. These findings are consistent in suggesting that possible sarcopenia is prevalent in the elderly, yet it has not yet received much attention.

Further, our study found that those with possible sarcopenia had a higher likelihood of recovering to non‐sarcopenia than deteriorating to sarcopenia, with a transition intensity 2.8 times greater. Given the notable proportion of possible sarcopenia in the geriatric population, coupled with a tendency for recovery, there arises a pending question as to the potential for over‐diagnosing this condition using current diagnostic criteria or functional cut‐off points. Trevisan et al. previously investigated transitions through sarcopenia stages based on MSM model over a 12‐year follow‐up period in 3219 older adults aged ≥60 years residing in Sweden, using EWGSOP2 criteria.^17^ When considering the estimated transition probabilities over a 5‐year period for individuals, their study reported a probability of transitioning from possible sarcopenia to non‐sarcopenia at 0.107, whereas ours was relatively higher at 0.322. The probabilities of maintaining possible sarcopenia (0.343 vs. 0.389) and progressing to sarcopenia (0.103 vs. 0.132) were similar. These differences in outcomes may be attributed to various factors. Specifically, our study comprised a relatively lower proportion of females (64.2% vs. 49.2%) and a younger mean age (74.2 ± 10.96 years vs. 68.5 ± 6.5 years) compared to theirs, as our results revealed a lower likelihood of recovery transition among females and older individuals. Another potential reason for the differences in transition probabilities is the different criteria for sarcopenia. AWGS2019 criteria, being more inclusive than EWGSOP2, may overestimate percentage of possible sarcopenia in the population, leading to higher recovery transition. In our study, 46% of participants had possible sarcopenia at baseline, while Trevisan et al. reported 27% with possible sarcopenia. The omission of diagnosing possible sarcopenia as a premorbid condition in clinical practice can result in both underdiagnosis and undertreatment, whereas excessive diagnosis may lead to unnecessary utilization of healthcare resources. Further research is necessary to determine the optimal threshold for the diagnosis of possible sarcopenia in the geriatric population. Additionally, establishing globally standardized diagnostic criteria for sarcopenia is essential to enhance comparability in international research.

Our study identified potential transitions in sarcopenia states within a relatively short observation period. Recent evidence shows rapid onset sarcopenia in older adults. A study on initially non‐sarcopenic individuals aged 70–84 years found that 13.5% of males and 11.7% of females developed sarcopenia during a 2‐year follow‐up period.^18^ Another study on 1117 Chinese dialysis patients, 24.8% transitioned from non‐sarcopenia to sarcopenia over a 1‐year follow‐up period, while 24.4% experienced a reversal from sarcopenia to non‐sarcopenia.^19^ Although sarcopenia is commonly perceived as a progressive process, the concept of acute sarcopenia has recently been proposed by EWGSOP2, referring to muscle loss occurring within 6 months.^2^ The occurrence of acute sarcopenia after events such as exacerbation of chronic diseases, hospitalization, and surgeries have been observed.^20^, ^21^ For monitoring sarcopenia states in older individuals, particularly those who are advanced in age, have low body weight, or experience functional impairments, more frequent assessments may be necessary to capture the dynamic changes in this condition.

We evaluated the effects of covariates on sarcopenia state transitions. Age is the most important factor in the development of sarcopenia,^22^ we found that the likelihood of recovery transition of possible sarcopenia decreases with age gradually. Sarcopenia, characterized by muscle loss, includes both a decrease in the size and the number of muscle fibres, which is believed to be secondary to the death of motor neurons and denervation of muscle fibres, while atrophy is a consequence of protein degradation pathways.^23^, ^24^ The mechanisms implicated in sarcopenia involve neurologic factors related to the loss of motor neurons, endocrine changes such as decreased or loss of hormone expression (e.g., testosterone or growth hormone), loss of muscle motor units, as well as nutrition and lifestyle changes associated with sedentary habits.^24^ These molecular process include loss of proteostasis, impaired mitochondrial function, neuromuscular remodelling, inflammation, apoptotic signalling, among others,^23^, ^25^, ^26^ which are core hallmarks of aging.^27^ These factors may contribute to the decline in muscle resilience. Targeting age‐related mechanism may be a potential avenue for exploring the treatment of sarcopenia. The different worsening and recovery risk with age suggest that measures like exercise to promote resilience^28^ should be started early. Underweight as a risk factor for sarcopenia has been discussed.^29^, ^30^ However, we found that underweight individuals exhibit a higher likelihood of both recovery and worsening compared with those of normal weight. Additionally, the underweight group had a remarkably lower estimated possibility of observing possible sarcopenia states remaining unchanged than the normal and overweight/obese groups. Underweight individuals in possible sarcopenia state exhibit a high degree of instability, are prone to state transitions, and should be a priority population for monitoring and intervention. A study has found that excessive energy intake is associated with increased body weight and SMM in underweight geriatric stroke patients,^31^ whether dietary modifications to promote weight gain in underweight older adults may improve the recovery transition of sarcopenia needs to be further clarified. Moreover, our finding that any degree of physical functional impairment predisposes the transition from non‐sarcopenia to possible sarcopenia aligns with prior research, indicating that functional impairment increases the risk of developing sarcopenia.^29^ In addition, mild physical impairments hindered the recovery transition of sarcopenia, while severe impairments increased the risk of death transition. Active screening for possible sarcopenia is crucial in all individuals with any degree of impairment. Those with severe impairment have a higher likelihood of possible sarcopenia or sarcopenia transitioning to death and require close monitoring.

Other factors including sex, smoking, hypertension, and diabetes also show impacts on the bidirectional transitions of sarcopenia states. We found that women not only had lower risk of deteriorating transitions between sarcopenia states than men but also had a lower possibility of recovery transition between sarcopenia states. Studies have shown that older men are at a significantly higher risk of experiencing a reduction in skeletal muscle mass and strength, as well as a higher prevalence of sarcopenia compared to women,^32^ which is consistent with our finding that women were at a lower risk of worsening transition to sarcopenia states. However, we found that women were more unlikely to recover from sarcopenia than men. A possible explanation is the sex differences in the adaptive changes of muscle strength and size among older adults. Specifically, older men exhibited greater absolute increases in upper and lower limb strength as well as muscle mass in response to resistance exercise compared to older women.^32^ Moreover, in sarcopenia diagnostic criteria, grip strength is used as the assessment of muscle strength. Men with high upper body muscle strength and grip strength^33^ may show a more significant absolute increase in muscle strength values, making male improvements in muscle strength more easily detected by grip strength testing. Sex‐specific resistance training programming may be necessary to achieve recovery transition of sarcopenia transition. Longer duration of resistance training interventions and higher frequency of exercises contributed to the increase in upper extremity strength in older women,^32^ so for older women with sarcopenia, more active resistance training focused on higher weekly repetitions and duration of interventions maybe a reasonable recommendation. Our study results also demonstrate an association between co‐morbidities and a lower likelihood of transitioning from possible sarcopenia to non‐sarcopenia. Older adults with multiple co‐morbidities should be closely monitored for sarcopenia states, and proactive interventions may be beneficial in facilitating their recovery from possible sarcopenia and reducing mortality rates.

At present, limited intervention studies have been conducted for individuals with possible sarcopenia, with a primary focus on sarcopenia interventions. Exercise and nutritional support have been identified as the most important interventions for sarcopenia [S10]. A meta‐analysis by Shen et al. showed that muscle function can be improved through physical exercise in older adults with sarcopenia, particularly resistance training, which significantly improves muscle strength and physical performance.^34^ Progressive resistance training is a commonly used mode of resistance training that can increase muscle cross‐sectional area and grip strength in older adults.^35^ A resistance training programme of two weekly exercise sessions, incorporating upper‐ and lower‐body exercises performed with high effort for 1–3 sets of 6–12 repetitions, can be an effective prescription for sarcopenia.^36^ Despite the consistent evidence of resistance exercise effectiveness, optimizing exercise parameters remains a significant problem that needs to be addressed.^37^ It has also been found that adding nutritional intervention to exercise has a greater impact on grip strength than exercise alone.^34^ Increased protein or amino acid intake is still the most common dietary modality for sarcopenia.^37^, ^38^ The nutritional consensus for sarcopenia recommends a daily protein intake of ≥1.2 g/kg body weight for those with sarcopenia and/or frailty.^38^ Apart from specific nutrients, dietary patterns such as the Mediterranean diet may have a ‘myoprotective’ effect and a Westernized diet pattern may increase the risk of poor physical function.^39^ In addition, the transitions of sarcopenia states among the elderly population may be attributed to changes in hydration status. Older adults with sarcopenia have significantly lower daily water intake compared with those without sarcopenia.^40^ A recent study conducted a cross‐sectional investigation on the relationship between hydration status and sarcopenia in 190 community‐dwelling elderly individuals. Although plasma osmolality was not significantly associated with sarcopenia, body water content assessed through bioimpedance analysis (BIA) revealed that sarcopenic individuals had lower percentages of intracellular water and intracellular water/free fat mass ratio [S11]. In‐depth exploration of the details concerning dietary patterns, exercise routines, and medication history in future research may contribute to a more profound understanding of the recovery state transitions observed in sarcopenia.

This study also has several limitations. Firstly, the population in this study was an Asian geriatric population and the diagnosis of sarcopenia was based on Asian criteria; therefore, further validation is necessary for generalizing the findings. Secondly, the ASM for muscle mass was calculated based on an anthropometric equation, as assessing ASM using DXA in a large population sample can be difficult and costly to implement and the CHARLS design did not include calf circumference measurements. Although this anthropometric equation has been confirmed to align with ASM measured by DXA in the Chinese population,^11^ it is important to acknowledge the possibility of potential bias. In addition, in CHARLS Wave 1–Wave 3, only a randomly selected subsample of approximately 40% of participants received the physical exercise questionnaire,^8^ so we failed to include the physical exercise information as a covariate and further studies may be needed to investigate the effect of physical exercise on sarcopenia states transitions.

## Conclusions

Our study emphasized the bidirectional transitions of sarcopenia in older adults in a natural course. Possible sarcopenia as a premorbid condition is prevalent in the geriatric population and manifests the potential for recovery. Factors including age, BMI, and physical function impact the bidirectional transitions of sarcopenia. Further optimizing diagnostic thresholds and exploring targeted interventions for possible sarcopenia may provide benefits for older adults.

## Funding

This work was funded by the National Natural Science Foundation of China (Grant No. 81971033 and No. 82371427), Natural Science Foundation of Chongqing, China (CSTB2023NSCQBHX0018 and CSTB2023NSCQ‐MSX0323), and Kuanren Talents Program of the Second Affiliated Hospital of Chongqing Medical University (Grant No. kryc‐lj‐2105).

## Conflict of interest

The authors declared no potential conflicts of interest with respect to the research, authorship, and/or publication of this article.

## Supporting information


**Table S1** The comparison of characteristics in Wave1 between enrolled individuals and excluded individuals due to lacking follow‐up of sarcopenia states.
**Table S2** The distribution of missing values among all records of enrolled individuals.
**Table S3** The distribution of sarcopenia states in each wave.
**Table S4** Observed numbers of sarcopenia states transitions from one follow‐up to next follow‐up.
**Table S5** Multivariate MSM model of sarcopenia state transitions.
**Table S6** The estimated mean sojourn time before the next transition and predicted total stay of each sarcopenia transient state among different subgroups.
