# Supplementary material for: Bidirectional transitions of sarcopenia states in older adults: The longitudinal evidence from CHARLS
Source: J Cachexia Sarcopenia Muscle. 2024 Jul 12;15(5):1915–29. doi: 10.1002/jcsm.13541 (PMC11446714; doi:10.1002/jcsm.13541)
Supplement: Supplementary file 1 — Table S1 The comparison of characteristics in Wave1 between enrolled individuals and excluded individuals due to lacking follow‐up of sarcopenia states. Table S2 The distribution of missing values among all records of enrolled individuals. Table S3 The distribution of sarcopenia states in each wave. Table S4 Observed numbers of sarcopenia states transitions from one follow‐up to next follow‐up. Table S5 Multivariate MSM model of sarcopenia state transitions. Table S6 The estimated mean sojourn time before the next transition and predicted total stay of each sarcopenia transient state among different subgroups. [file JCSM-15-1915-s001.docx]

**Title:** Bidirectional transitions of sarcopenia states in older adults: the longitudinal evidence from CHARLS

**Authors**

Ya-Xi Luo,^1^, Xiao-Han Zhou ^1^, Tian Heng ^1^, Ling-Ling Yang^1^, Ying-Hai Zhu ^1^, Peng Hu^1^, Xiu-Qing Yao ^1,2,3^

**Author address**

1. Department of Rehabilitation, The Second Affiliated Hospital of Chongqing Medical University, Chongqing, China.

2. Chongqing Municipality Clinical Research Center for Geriatric Medicine, Chongqing, China.

3. Department of Rehabilitation Therapy, Chongqing Medical University, Chongqing, China.

**Address correspondence to**: Xiu-Qing Yao, Department of Rehabilitation, The Second Affiliated Hospital of Chongqing Medical University, Chongqing, China. Tel: +86 (23) 63693883. Email: [dryaoxq@cqmu.edu.cn](mailto:dryaoxq@cqmu.edu.cn)

**Supplemental materials**

**Supplementary Table.1** **The comparison of characteristics in Wave1 between enrolled individuals and excluded individuals due to lacking follow-up of sarcopenia states**

| **Demographic and Clinical Characteristics** | **Enrolled**  **(n = 4395)** | **Excluded**  **(n = 2895)** | ***P* value** |
| --- | --- | --- | --- |
| **Age (median, IQR)** | 67 (63-72) | 66 (62-73) | 0.174 |
| **Sex (n, %)** |  |  | 0.219 |
| Female | 2161 (49.2) | 1466 (50.6) |  |
| Male | 2234 (50.8) | 1429 (49.4) |  |
| **Marital status (n, %)** |  |  | 0.349 |
| Married/partnered | 3430 (78.0) | 2230 (77.0) |  |
| Divorced/separated/widowed/never married | 965 (22.0) | 662 (22.9) |  |
| Missing(n) | 0 | 3 |  |
| **Education (n, %)** |  |  | <0.001 |
| Below high school level | 4203 (95.6) | 2579 (89.1) |  |
| At or above high school level | 192 (4.4) | 313 (10.9) |  |
| Missing(n) | 0 | 3 |  |
| **Body mass index (mean, SD)** | 23.23 (27.30) | 23.93 (17.384) | 0.242 |
| Missing(n) | 350 | 1159 |  |
| **ADL score (median, IQR)** | 0 (0-1) | 0 (0-0) | 0.736 |
| Missing (n) | 41 (0.9) | 75 (2.6) |  |
| **CESD-10 score (median, IQR)** | 8 (4-14) | 7 (3-13) | **<0.001** |
| Missing (n) | 180 (4.1) | 418 (14.4) |  |
| **Smoking (n, %)** |  |  | **<0.001** |
| No | 2993 (69.3) | 2003 (73.7) |  |
| Yes | 1326 (30.7) | 713 (26.3) |  |
| Missing (n) | 76 | 179 |  |
| **Alcohol consumption (n, %)** |  |  | 0.171 |
| No | 3049 (69.6) | 2025 (71.1) |  |
| Yes | 1332 (30.4) | 823 (28.9) |  |
| Missing(n) | 14 | 47 |  |
| **Hypertension (n, %)** |  |  | **0.010** |
| No | 2916 (66.8) | 1815 (63.9) |  |
| Yes | 1449 (33.2) | 1027 (36.1) |  |
| Missing(n) | 30 | 53 |  |
| **Diabetes Mellitus (n, %)** |  |  |  |
| No | 4039 (92.8) | 2591 (91.7) | 0.080 |
| Yes | 313 (7.2) | 235 (8.3) |  |
| Missing(n) | 43 | 69 |  |
| **Pulmonary diseases (n, %)** |  |  | 0.074 |
| No | 3747 (85.8) | 2476 (87.2) |  |
| Yes | 622 (14.2) | 362 (12.8) |  |
| Missing(n) | 26 | 57 |  |
| **Cardiac diseases (n, %)** |  |  | **<0.001** |
| No | 3710 (85.1) | 2302 (81.3) |  |
| Yes | 651 (14.9) | 529 (18.7) |  |
| Missing(n) | 34 | 64 |  |
| **Cancer history (n, %)** |  |  |  |
| No | 4325 (99.2) | 2805 (98.8) | 0.116 |
| Yes | 36 (0.8) | 34 (1.2) |  |
| Missing(n) | 34 | 56 |  |
| **Stroke history (n, %)** |  |  | 0.517 |
| No | 4191 (95.8) | 2716 (95.5) |  |
| Yes | 183 (4.2) | 128 (4.5) |  |
| Missing(n) | 21 | 51 |  |

Note: data were summarized as medians with interquartile ranges (IQRs), means± standard deviations (SDs), and numbers (%) for skewed distributed continuous variables, normally distributed continuous variables, and categorical variables, respectively, and compared using the Mann-Whitney U test, Student’s t-test, chi-square test, as appropriate. Abbreviations: ADL, Activities of Daily Living; CESD: Center for epidemiologic studies depression scale.

**Supplementary Table.2 The distribution of missing values among all records of enrolled individuals.**

| **Distribution s of missing value, n (%)** | **Total records = 10778** |
| --- | --- |
| **Age** | 0 (0) |
| **Sex** | 0 (0) |
| **Marital status** | 0 (0) |
| **Education** | 0 (0) |
| **Body mass index** | 671 (6.2) |
| **ADL score** | 533 (4.9) |
| **CESD10 score** | 944 (8.8) |
| **Smoking** | 1420 (13.2) |
| **Alcohol consumption** | 529 (4.9) |
| **Hypertension** | 395 (3.7) |
| **Diabetes Mellitus** | 604 (5.6) |
| **Pulmonary diseases** | 458 (4.2) |
| **Cardiac diseases** | 516 (4.8) |
| **Cancer history** | 579 (5.4) |
| **Stroke history** | 523 (4.9) |

Abbreviations: ADL, Activities of Daily Living; CESD: Center for epidemiologic studies depression scale.

**Supplementary Table.3 The distribution of sarcopenia states in each wave**

|  | **Non-sarcopenia** | **Possible sarcopenia** | **Sarcopenia** | **Death** | **Total records** |
| --- | --- | --- | --- | --- | --- |
| **Wave 1, n (%)** | 1373 (36.4) | 1733 (46.0) | 662 (17.6) | 0 (0) | 3768 |
| **Wave 2, n (%)** | 1361 (39.1) | 1388 (39.9) | 530 (15.2) | 204 (5.9) | 3483 |
| **Wave 3, n (%)** | 1259 (35.7) | 1390 (39.4) | 581 (16.5) | 297 (8.4) | 3527 |

**Supplementary Table.4 Observed numbers of sarcopenia states transitions from one follow-up to next follow-up**

|  | | **Post-transition, n (%)** | | | |
| --- | --- | --- | --- | --- | --- |
|  |  | **Non-sarcopenia** | **Possible Sarcopenia** | **Sarcopenia** | **Death** |
| **Pre-transition,**  **n (%)** | **Non-sarcopenia** | 1516 (61.5) | 671 (27.2) | 177 (7.2) | 103 (4.2) |
|  | **Possible sarcopenia** | 693 (24.5) | 1705 (60.3) | 189 (6.7) | 239 (8.5) |
|  | **Sarcopenia** | 156 (14.3) | 121 (11.1) | 654 (60.0) | 159 (14.6) |

**Supplementary Table.5 Multivariate MSM model of sarcopenia state transitions**

| **Hazard Ratio (95% CI)** | | | | | | | | | | | | | | |
| --- | --- | --- | --- | --- | --- | --- | --- | --- | --- | --- | --- | --- | --- | --- |
| **Covariates** | **Deteriorate transition** | | | | **Recovery transition** | | | | **Death transition** | | | | |  |
|  | **Non-sarcopenia→**  **Possible sarcopenia** | | **Possible sarcopenia→ Sarcopenia** | | **Sarcopenia→**  **Possible sarcopenia** | | **Possible sarcopenia→**  **Non-sarcopenia** | | **Non-sarcopenia→**  **Death** | **Possible sarcopenia →Death** | | **Sarcopenia→ Death** | |  |
| **Age** | |  | |  | |  | |  |  | |  | |  | |
| 60-70 | | Reference | |  | | Reference | |  | Reference | |  | |  | |
| 71-80 | | **1.417**  **(1.161-1.730)** | | **2.019**  **(1.447-2.815)** | | 0.740  (0.478-1.146) | | **0.499**  **(0.397-0.626)** | 1.885  (0.668-5.322) | | 0.729  (0.354-1.504) | | **2.391**  **(1.255-4.555)** | |
| ＞80 | | 1.086  (0.561-2.101) | | **1.993**  **(1.167-3.405)** | | **0.358**  **(0.187-0.685)** | | **0.113**  **(0.050-0.258)** | **8.259**  **(1.333-51.162)** | | **3.328**  **(1.775-6.238)** | | **3.249**  **(1.598-6.606)** | |
| **Sex** | |  | |  | |  | |  |  | |  | |  | |
| Male | | Reference | |  | | Reference | |  | Reference | |  | |  | |
| Female | | 1.066  (0.871-1.306) | | 1.196  (0.861-1.661) | | 0.976  (0.652-1.462) | | **0.775**  **(0.629-0.956)** | 1.557  (0.304-7.963) | | **0.390**  **(0.211-0.715)** | | 0.726  (0.505-1.045) | |
| **Body mass index** | |  | |  | |  | |  |  | |  | |  | |
| Normal | | Reference | |  | | Reference | |  | Reference | |  | |  | |
| Underweight | | **1.579**  **(1.001-2.492)** | | 244.704  (0.169,3.546e+05) | | 16.212  (0.010-2.513e+04) | | **5.174**  **(2.856-9.372)** | 0.335  (0.003-35.190) | | 0.797  (0.001-4.731e+02) | | 1.004  (0.703-1.433) | |
| Overweight/obese | | **0.801**  **(0.662-0.969)** | | **0.257**  **(0.110-0.597)** | | 0.750  (0.005-123.740) | | 0.854  (0.701-1.043) | 0.706  (0.260-1.915) | | 0.778  (0.445-1.358) | | **8.740**  **(4.897-15.597)** | |
| **Physical function** | |  | |  | |  | |  |  | |  | |  | |
| No impairment | | Reference | |  | | Reference | |  | Reference | |  | |  | |
| Mild impairment | | 1.399  (1.111-1.761) | | 0.651  (0.460-0.921) | | 0.546  (0.359-0.832) | | 0.824  (0.643-1.057) | 2.152  (0.829-5.588) | | 1.099  (0.605-1.995) | | 1.225  (0.859-1.746) | |
| Severe impairment | | 2.003  (1.295-3.099) | | 1.219  (0.778-1.909) | | 0.838  (0.433-1.623) | | 0.776  (0.481-1.253) | 2.662  (0.534-13.278) | | 2.468  (1.443-4.222) | | 1.666  (1.087-2.553) | |
| **Smoking** | |  | |  | |  | |  |  | |  | |  | |
| No | | Reference | |  | | Reference | |  | Reference | |  | |  | |
| Yes | | 0.812  (0.647-1.020) | | **1.660**  **(1.152-2.392)** | | **1.737**  **(1.120-2.693)** | | 0.812  (0.644-1.023) | **7.575**  **(1.094-52.468)** | | 0.601  (0.299-1.207) | | 0.977  (0.666-1.433) | |
| **Hypertension** | |  | |  | |  | |  |  | |  | |  | |
| No | | Reference | |  | | Reference | |  | Reference | |  | |  | |
| Yes | | 0.970  (0.802-1.172) | | 0.796  (0.577-1.096) | | 1.3093  (0.8573-2.000) | | **0.707**  **(0.580-0.861)** | **3.143**  **(1.215-8.128)** | | **1.740**  **(1.043-2.905)** | | 1.226  (0.840-1.791) | |
| **Diabetes** | |  | |  | |  | |  |  | |  | |  | |
| No | | Reference | |  | | Reference | |  | Reference | |  | |  | |
| Yes | | 0.873  (0.639-1.191) | | 1.325  (0.704-2.495) | | 1.047  (0.315-3.486) | | **0.701**  **(0.502-0.980)** | 0.646  (0.067-6.228) | | 0.876  (0.293-2.616) | | **2.380**  **(1.267-4.470)** | |

Boldface indicates statistical significance (P<0.05).

**Supplementary Table.6 The estimated mean sojourn time before the next transition and predicted total stay of each sarcopenia transient state among different subgroups**

|  |  | **Estimated mean sojourn times in each transient state (year)** | | |  | **Predicted total length of stay of each transient state before death (year)** | | |
| --- | --- | --- | --- | --- | --- | --- | --- | --- |
|  |  | Non-sarcopenia | Possible sarcopenia | Sarcopenia |  | Non-  sarcopenia | Possible sarcopenia | Sarcopenia |
| **All individuals** | | 2.83 | 2.27 | 2.19 |  | 13.67 | 13.02 | 4.67 |
| **Age** | 60-70 | 3.34 | 1.63 | 3.09 |  | 26.60 | 20.98 | 4.83 |
|  | 71-80 | 2.35 | 2.22 | 3.28 |  | 6.09 | 10.10 | 5.53 |
|  | ＞80 | 2.97 | 2.93 | 4.32 |  | 2.56 | 4.48 | 2.40 |
| **Sex** | Male | 3.34 | 1.63 | 3.09 |  | 12.63 | 10.05 | 3.71 |
|  | Female | 3.13 | 1.99 | 3.27 |  | 15.37 | 17.84 | 6.24 |
| **Body mass index** | Normal | 3.34 | 1.63 | 3.09 |  | 12.90 | 12.43 | 5.44 |
|  | Underweight | 2.13 | 0.04 | 0.22 |  | 5.21 | 1.31 | 9.23 |
|  | Overweight/obese | 4.18 | 2.13 | 1.78 |  | 16.07 | 16.13 | 0.61 |
| **Physical function** | No impairment | 3.34 | 1.63 | 3.09 |  | 18.15 | 15.00 | 5.52 |
|  | Mild impairment | 2.38 | 2.00 | 4.91 |  | 8.83 | 13.08 | 3.77 |
|  | Severe impairment | 1.67 | 1.75 | 3.29 |  | 3.93 | 6.56 | 2.78 |
| **Smoking** | No | 3.34 | 1.63 | 3.09 |  | 12.41 | 13.09 | 4.59 |
|  | Yes | 3.93 | 1.76 | 1.88 |  | 17.17 | 13.01 | 5.00 |
| **Hypertension** | No | 3.34 | 1.63 | 3.09 |  | 16.21 | 13.61 | 6.45 |
|  | Yes | 3.40 | 2.10 | 2.38 |  | 10.36 | 12.07 | 2.45 |
| **Diabetes** | No | 3.34 | 1.63 | 3.09 |  | 13.98 | 13.08 | 4.95 |
|  | Yes | 3.84 | 2.03 | 2.55 |  | 10.85 | 12.22 | 2.21 |

**Additional references**

S1. Coletta G and Phillips SM. An elusive consensus definition of sarcopenia impedes research and clinical treatment: A narrative review. *Ageing research reviews* 2023; 86: 101883.

S2. Echouffo-Tcheugui JB and Selvin E. Prediabetes and What It Means: The Epidemiological Evidence. *Annual review of public health* 2021; 42: 59-77.

S3. Bloom I, Shand C, Cooper C, et al. Diet Quality and Sarcopenia in Older Adults: A Systematic Review. *Nutrients* 2018; 10.

S4. Hurst C, Robinson SM, Witham MD, et al. Resistance exercise as a treatment for sarcopenia: prescription and delivery. *Age and ageing* 2022; 51

S5. von Elm E, Altman DG, Egger M, et al. The Strengthening the Reporting of Observational Studies in Epidemiology (STROBE) statement: guidelines for reporting observational studies. *Lancet (London, England)* 2007; 370: 1453-1457.

S6. Gao K, Cao LF, Ma WZ, et al. Association between sarcopenia and cardiovascular disease among middle-aged and older adults: Findings from the China health and retirement longitudinal study. *EClinicalMedicine* 2022; 44: 101264.

S7. Guralnik JM, Simonsick EM, Ferrucci L, et al. A short physical performance battery assessing lower extremity function: association with self-reported disability and prediction of mortality and nursing home admission. *Journal of gerontology* 1994; 49: M85-94.

S8. Greysen SR, Stijacic Cenzer I, Auerbach AD, et al. Functional impairment and hospital readmission in Medicare seniors. *JAMA internal medicine* 2015; 175: 559-565.

S9. Vellas B, Fielding RA, Bens C, et al. Implications of ICD-10 for Sarcopenia Clinical Practice and Clinical Trials: Report by the International Conference on Frailty and Sarcopenia Research Task Force. *The Journal of frailty & aging* 2018; 7: 2-9.

S10. Negm AM, Lee J, Hamidian R, et al. Management of Sarcopenia: A Network Meta-Analysis of Randomized Controlled Trials. *Journal of the American Medical Directors Association* 2022; 23: 707-714. 2022/02/21.

S11. Serra-Prat M, Lorenzo I, Martinez J, et al. Relationship between Hydration Status and Muscle Catabolism in the Aged Population: A Cross-Sectional Study. Nutrients. 2023;15(22).
